# Trauma-Informed Care on mental health wards: the impact of Power Threat Meaning Framework Team Formulation and Psychological Stabilisation on self-harm and restrictive interventions

**DOI:** 10.3389/fpsyg.2023.1145100

**Published:** 2023-06-08

**Authors:** Faye Nikopaschos, Gail Burrell, Jordan Clark, Ana Salgueiro

**Affiliations:** Harrow Mental Health, Central and North West London NHS Foundation Trust (CNWL), London, United Kingdom

**Keywords:** Trauma-Informed Care (TIC), Power Threat Meaning Framework (PTMF), stabilisation, self-harm, restrictive intervention, seclusion, restraint, inpatient

## Abstract

**Aim:**

The aim of this evaluation was to assess the impact of introducing a model of Trauma-Informed Care (TIC), comprising weekly Power Threat Meaning Framework (PTMF) Team Formulation and weekly Psychological Stabilisation staff training, to a National Health Service (NHS) adult acute inpatient mental health unit over a four-year period.

**Method:**

A retrospective service evaluation design was employed to assess for differences in the number of incidents of self-harm, seclusion and restraint in the four-year period following the introduction of TIC, when compared to the year prior.

**Results:**

Significant reductions were demonstrated in the monthly number of incidents of self-harm (*p* < 0.01; r = 0.42), seclusion (*p* < 0.05; r = 0.30) and restraint (*p* < 0.05; d = 0.55) following the introduction of TIC.

**Conclusion:**

Findings suggest that PTMF Team Formulation and Psychological Stabilisation training can contribute to significant reductions in self-harm and restrictive interventions (seclusion and restraint) on adult mental health wards. Qualitative interviews with staff and service users from the unit will support a better understanding of the mechanisms of this change. Further research, employing a randomised control trial design, could increase the validity and generalisability of findings. However, the ethical implications of withholding potentially beneficial practices from a control group would need to be considered.

## 1. Introduction

A large body of evidence demonstrates the relationship between experiences of trauma and adversity, and the subsequent development of mental health difficulties (or human distress). Research consistently shows high rates of traumatic and adverse experiences in people using mental health services (Kessler et al., 2010; Khalifeh et al., 2016) and these are greater than the levels found within the general population (Mauritz et al., 2013). Experiencing trauma in childhood, in particular childhood sexual abuse, has been strongly linked to the development of difficulties commonly labelled as psychosis (Bebbington et al., 2011; Varese et al., 2012). Childhood trauma has also been linked to both manic and depressive states (Putman, 2003; Aas et al., 2016) as well as difficulties associated with a diagnosis of personality disorder (Porter et al., 2020). Diagnoses of anxiety and depression are commonly linked with a range of traumatic and adverse experiences occurring in both child and adulthood (Campbell, 2002; Kendler et al., 2003). Wider social factors such as poverty, racism and violence are also strongly correlated with poor mental health (Murali and Oyebode, 2004; Paradies, 2006). It is notable that the impact of trauma and adversity on mental health has been shown to be cumulative, with the severity, frequency and range of adverse experiences all associated with subsequent mental health impact (Shevlin et al., 2008; Dillon et al., 2012).

This research clearly demonstrates the necessity for services and clinicians to foreground the impact of trauma and adversity in their approach to understanding mental health difficulties and delivering appropriate intervention. In line with this, the NHS Long Term Plan (NHS, 2019) has laid out recommendations for mental health services to become trauma-informed over the next 10 years.

Trauma-Informed Care (TIC) *realises* the impact of trauma, *recognises* the signs and symptoms of trauma, *responds* by integrating knowledge about trauma into its practice, and seeks to actively *resist re-traumatisation* (Huang et al., 2014). A developing evidence-base demonstrates the benefits of TIC in mental health settings. Research has shown that TIC can reduce levels of distress/ symptoms (Messina et al., 2014) and facilitate improved coping skills (Gatz et al., 2007). Within inpatient mental health services specifically, TIC has been shown to reduce restrictive intervention (Azeem et al., 2011); improve relationships between staff and service users (Chandler, 2008); reduce length of admission; and improve service users' mental wellbeing (Greenwald et al., 2012). Research describes a range of practices utilised to support TIC, including staff training (Azeem et al., 2011) and psychological safety-based interventions (Gatz et al., 2007). However, as yet, evidence-based recommendations operationalising this approach for mental health services have not been developed.

From a trauma-informed perspective, recovery is conceptualised as having three key stages (Herman, 2015). The establishment of safety, also known as ‘Stabilisation', is seen as both primary and essential for overcoming trauma-related difficulties within this model. Stabilisation can take many different forms, including social (e.g., access to food, finances, housing), physical (e.g., medication, physical healthcare) and psychological. Psychological Stabilisation is the process of learning and putting into practice skills to support internal emotional safety, and a range of therapy models draw upon these principles (Linehan, 2014; National Institute for Health Clinical Excellence, 2018). Subsequent stages of recovery, which do not proceed in a strictly linear manner, are “Trauma Processing” (for example, through therapy) and “Reconnection” with oneself and one's life (Herman, 2015). From a trauma-informed perspective, it is of particular importance that safety (the “Stabilisation” phase) is established before work directly relating to an individual's experience/s of trauma is approached.

One practice that has been used to support shifts in culture towards more trauma-informed understandings of distress is Team Formulation (Cole et al., 2015; Dexter-Smith, 2015). Johnstone (2013) describes Team Formulation as “the process of facilitating a group or team of professionals to construct a shared understanding of a service user's difficulties”. Benefits of this practice include, enhanced psychological thinking and increased positive attitudes toward service users (Division of Clinical Psychology, 2011); greater understanding of risk (Ramsden et al., 2014); and improved relationships between staff and service users (Berry et al., 2015). The practice of Team Formulation varies, and can be facilitated in a range of ways, which are not all necessarily trauma-informed.

The Power Threat Meaning Framework (PTMF; Johnstone and Boyle, 2018) is an alternative conceptual approach to the traditional diagnostic model of mental health, co-produced by professionals and service users, and published by the British Psychological Society. It draws upon a wide range of perspectives, including TIC, and offers an evidence-based meta-framework that can be used to support trauma-informed understanding of mental health difficulties. Six core questions are designed to support narrative construction, as follows: (1) What has happened to you? (How is power operating in your life?); (2) How did it affect you? (What kind of threats does this pose?); (3) What sense did you make of it? (What is the meaning of these situations and experiences to you?); (4) What did you have to do to survive? (What kind of threat response/s are you using?); (5) What are your strengths? (What access to power resources do you have?); and (6) What is your story? (How does this fit together?).

In line with the recommendations of the NHS Long Term Plan (NHS, 2019) and the evidence-base demonstrating the relationship between trauma, adversity and mental health difficulties, a model of TIC was developed and introduced to an adult inpatient mental health unit over a four-year period. The model comprised two practices to support trauma-informed formulation (PTMF Team Formulation) and intervention (Psychological Stabilisation). This evaluation aims to assess the impact of these practices.

## 2. Materials and methods

### 2.1. Design

This retrospective service evaluation is based on quantitative data collected from an NHS adult acute inpatient mental health unit (consisting of two wards) at a North London Hospital over a five-year period (July 2017–June 2022). Data from the first year of the evaluation (July 2017–June 2018) and prior to the introduction of Trauma-Informed Care (TIC) was compared with data from the subsequent four years (July 2018–June 2022) following the introduction of TIC.

This paper is part of a larger project evaluating the impact of TIC at the inpatient unit and involving the analysis of both quantitative and qualitative outcome data. Due to the scope of this paper, only the quantitative data will be reported. Qualitative data from interviews with staff and service users illustrate some of the mechanisms of change, and will be analysed and written up for later publication.

The project was registered with the NHS Trust's (Central and North West London NHS Foundation Trust [CNWL]) Information and Governance Team. A Data Protect Impact Assessment (DIPA) was completed and approved by this team.

### 2.2. Setting

The mental health unit houses two adult acute wards, originally 23 and 22 bedded (July 2017) and gradually both reduced to 18 bedded (from October 2020 to January 2022) in line with National Institute for Health and Clinical Excellence (NICE, 2015) Safe Staffing Standards. Adults are admitted following assessment in Accident and Emergency or the community via the crisis pathway, and may self-present or be referred by statutory services, family and friends, or the police. Admissions may be Informal or under Section of the Mental Health Act (the UK legislation, which covers the assessment, treatment and rights of people with mental health difficulties).

The inpatient clinical team is multi-disciplinary. Each ward is staffed 24/7 by a nursing team, comprising 2 qualified nurses and 3 health care assistants per shift. Over the period of this evaluation, the day team (Monday–Friday; 9 am−5 pm) for each ward consisted of 1 whole time equivalent (WTE) Consultant Psychiatrist, 1.6 WTE Specialty Doctors and 2 WTE Junior Doctors, 2 WTE Occupational Therapists, 1 WTE Peer Support Worker, 0.5 WTE Fitness Instructor and 0.4 WTE Psychologist.

### 2.3. Procedure

From July 2018 onwards a model of TIC, comprising two practices that support trauma-informed formulation and intervention, was developed and introduced to the inpatient mental health unit. These practices are described below.

#### 2.3.1. Trauma-informed practice 1: Power Threat Meaning Framework Team Formulation

A model of trauma-informed Team Formulation, informed by the Power Threat Meaning Framework (PTMF), was developed. This practice was delivered to the inpatient multi-disciplinary team (MDT) in a one-hour weekly meeting (alternating fortnightly between the two wards). Meetings were facilitated by two of four staff (TIC Champions) who were all senior MDT members. They included the Principal Psychologist, the Deputy Borough Director (who is an Occupational Therapist by background), the Lead Occupational Therapist and Matron. The meetings were structured in accordance with a standard Protocol (see Appendix 1) and Quality Measure (available from the authors), which were drawn up to ensure consistency and attendance to all key areas.

The full MDT was invited to attend the meeting, which is designed to serve as a form of staff supervision/ consultation. In line with the Association of Clinical Psychologists' (ACP, 2022) Guidance, service users were involved in several ways. Although not physically present in the meeting, where possible, the service user was informed in advance, asked for consent, and invited to say what they felt was important for the team to understand and think about during the discussion. Feedback was given to the service user following the meeting by one or two key professionals from the team. CNWL service users (with a previous history of admission/s to the inpatient wards) were also consulted in the development of the Protocol for the Team Formulation structure and process.

#### 2.3.2. Trauma-informed practice 2: Psychological Stabilisation training

A Stabilisation Manual was developed by the authors (Nikopaschos et al., 2020) comprising one introductory session and ten intervention sessions (1. Self-Compassion; 2. Soothing & Safety; 3. Mindfulness; 4. Effective Communication; 5. Breathing & Relaxation; 6. Food & Sleep; 7. Distraction & Distancing; 8. Valued Activity; 9. Grounding; 10. Maintaining Wellbeing). The manual was based on the Cwm Taf Morgannwg University Health Board Psychological Therapies Department (2017) Stabilisation Pack and draws on a range of established strategies and skills for first-stage trauma work (Linehan, 2014). For further information, the full manual is freely accessible online (see references).

A corresponding staff training program was designed to enable the delivery of the Stabilisation Manual interventions to inpatients in planned individual and group sessions, as well as to support immediate de-escalation and distress management on the wards. The training was delivered to the full multi-disciplinary team (MDT) on both wards on a rolling weekly one-hour basis and facilitated by the TIC Champions (the same senior staff group who facilitated PTMF Team Formulation). Each session was discrete and corresponded to one session from the manual. The Stabilisation Manual was also available for all inpatients to work through self-guided.

### 2.4. Measures

The following demographic and clinical variables were extracted from the electronic clinical notes system for each episode of inpatient care (admission) over the study period; age, ethnicity, gender, legal status (Informal or admitted under the Mental Health Act), and diagnosis. Staff attendance at PTMF Team Formulation and Stabilisation training was recorded using a session-by-session register.

In line with both the ideological position of TIC (to resist staff practices and service user experiences that hold the potential for re-traumatisation) as well as the NHS Trust's priorities to monitor and reduce incidents of self-harm and the use of restrictive intervention (seclusion and restraint) on inpatient wards, the unit's electronic incident reporting system (Datix) was utilised to gather data on the frequency of the following incidents over the evaluation period. 1. Self-harm (any intentional act of harm to the self); 2. seclusion (the supervised confinement and isolation of an inpatient to an area from which they are prevented from leaving); and 3. restraint (any direct physical contact, from a staff member to an inpatient, where the intention is to prevent, restrict of subdue movement of the body). These were also the incident types that appeared to show variation in reporting frequency over the evaluation period (when the unit's electronic incident reporting system was periodically reviewed) and therefore prioritised for further analysis.

Staff are required to report all incidents of self-harm, seclusion and restraint occurring at the unit by completing a Datix report. For incidents not observed by staff but reported to staff by service users, CCTV footage is reviewed. All incidents are routinely reviewed at the start and end of each shift as a safeguard to ensure the appropriate reporting of incidents via Datix. Inpatients are also given the opportunity to report incidents in weekly community meetings and daily 1:1 contact time with staff.

As the COVID-19 pandemic (April 2020 onwards) coincided with the evaluation period, the potential impact of this in terms of staff sickness absence was also measured by extracting the total number of staff sick days (COVID-19 and non COVID-19 related) for each of the five years of the evaluation period from the unit's staff absence reporting system.

### 2.5. Data analysis

Data was analysed using Microsoft Excel 2019 on a Windows PC. Descriptive statistics were utilised to assess for any variability in the number of inpatient admissions across the five years of the evaluation period (July 2017–June 2022). The number of incidents of self-harm, seclusion and restraint were calculated monthly for the five-year evaluation period (July 2017–June 2022). Initial comparisons were made between the monthly mean number of incidents of self-harm, seclusion and restraint for the one-year period prior to the introduction of TIC (July 2017–June 2018; hereafter referred to as pre-TIC) and the four-year period following the introduction of TIC (July 2018–June 2022; hereafter referred to as post-TIC). Parametric assumptions for this data were assessed using Shapiro-Wilk's test (for normality) and Levene's test (for homogeneity of variance). For variables where this assumption was met (restraint) an independent *t*-test was used to assess the statistical significance of differences in the monthly number of incidents pre- and post-TIC. Cohen's d was used to estimate the effect size. Where parametric assumptions were not met (self-harm, seclusion) Mann-Whitney's *U* test was utilised and Rosenthal's (1991) method employed to convert the Z-score into an effect size estimate (r). Statistical significance was set to p<0.05 and effect sizes interpreted using Funder and Ozer's (2019) classification for r, and Cohen's (1988) classification for d.

Further exploratory analysis was conducted by reviewing the four years of data for self-harm, seclusion and restraint post-TIC in yearly samples. Descriptive statistics (monthly mean, standard deviation, percentage of change in the monthly mean) were utilised to assess for differences in the three variables for the year pre-TIC with each of the four subsequent one-year time periods post-TIC in turn. Further formal statistical analysis at this level was not employed due to the modest sample size.

Finally, as the evaluation period coincided with the COVID-19 pandemic (April 2020 onwards), descriptive statistics (total number, percentage of change) were utilised to consider the potential impact of COVID-19 related staff sickness on variations seen in the restrictive interventions data, when analysed by year. Staff sick days were divided into COVID-19 (categorised as either a: staff COVID-19 positive or b: staff isolating due to household member with COVID-19) and non COVID-19 related sickness, and calculated for each of the five years of the evaluation period.

## 3. Results

### 3.1. Admissions

There were 2,287 new admissions to the mental health unit between July 2017 and June 2022. The number of inpatient admissions remained relatively consistent over the five-year evaluation period, as detailed in Table 1 below. The highest number of admissions was in Year 2 post-TIC (n=491) and the lowest in Year 4 post-TIC (*n* = 436).

**Table 1:** Number of inpatient admissions by year.

**Date range**	**Time period**	**Number of admissions**
Jul 2017–Jun 2018	Pre-TIC	474
Jul 2018–Jun 2019	Year 1 Post-TIC	445
Jul 2019–Jun 2020	Year 2 Post-TIC	491
Jul 2020–Jun 2021	Year 3 Post-TIC	441
Jul 2021–Jun 2022	Year 4 Post-TIC	436

In addition to the 2,287 new admissions to the unit over the period of evaluation, there were 45 inpatients already admitted to the unit on the 1st July 2017 (first date of the study period). This represents a total of 2,332 discrete episodes of inpatient care (over 1625 different inpatients) over the five-year evaluation period.

### 3.2. Demographics

Episodes of inpatient care were classified by the following demographic variables. 48.24% (*n* = 1,125) female; 51.50% male (*n* = 1,201); and 0.26% gender not specified (n=6). 15.91% under the age of 25 years (*n* = 371); 61.32% 25-49 years (*n* = 1,430); 22.51% 50–74 years (*n* = 525); and 0.25% (*n* = 6) over 75 years. In terms of ethnicity, 22.68% were Asian or Asian British (*n* = 529); 17.71% Black, Black British, Caribbean or African (*n* = 413); 3.09% mixed or multiple ethnic groups (*n* = 72); 29.25% White (n=682); and 27.27% other/ not stated (*n* = 636).

60.55% (*n* = 1412) episodes of inpatient care were under Section of the Mental Health Act and 39.45% (*n* = 920) were Informal. The presenting issue on admission was classified by the following diagnoses, 40.14% psychosis (*n* = 936); 9.73% bipolar affective disorder (*n* = 227); 10.51% depression/ anxiety (*n* = 245); 7.72% personality disorder (*n* = 180); 4.03% acute stress reaction (*n* = 94); 6.26% substance use (*n* = 146); 3.37% other (*n* = 102); and 17.24% no diagnosis recorded (*n* = 402).

### 3.3. Attendance

#### 3.3.1. Power Threat Meaning Framework Team Formulation

Weekly Team Formulation commenced in July 2018 and ran 147 times until the end of June 2022. The meeting was cancelled 58 times over this period due to staff shortages (29 times); national/ bank holidays (10 times); and COVID-19 outbreaks (19 times). There was a total of 1170 staff attendances at the meeting, with a mean weekly attendance of 8 staff per meeting, not including the 2 facilitators. Attendees included, nursing (46%); occupational therapy (24%); psychology (16%); psychiatry (8%); peer support (3%); and other staff (including administrators, fitness instructors, volunteers and pharmacy; 3%).

#### 3.3.2. Psychological Stabilisation training

Weekly Stabilisation training for the ward staff commenced in November 2018 and ran 119 times until the end of June 2022. Training was cancelled 69 times over this period due to staff shortages (45 times); national/ bank holidays (4 times); and COVID-19 outbreaks (20 times). There was a total of 706 staff attendances at the training, with a mean weekly attendance of 6 staff per meeting. These figures do not include the 2 facilitators. Attendees included nursing (48%); occupational therapy (20%); psychology (20%); peer support (8%); psychiatry (1%); social work (1%); and other staff (1%).

### 3.4. Self-harm

There was a total of 257 incidents of self-harm over the five-year evaluation period (July 2017–June 2022). 93 of these occurred in the first year of the evaluation (pre-TIC). This was compared to 164 in the four-year period post-TIC. Self-harm incidents were categorised as, cuts (*n* = 75; 29.18%); ligatures (*n* = 64; 24.90%); overdoses (*n* = 22; 8.56%); falling/ jumping from a height (*n* = 9; 3.50%); and other (*n* = 85; 33.85%).

The monthly mean number of self-harm incidents in the year pre-TIC was 7.75. This was compared to a monthly mean of 3.42 self-harm incidents in the four-year period post-TIC. This represents an overall reduction of 55.91% in self-harm on the inpatient wards post-TIC. Statistical analysis suggests this reduction to be significant with a very large effect size (see Tables 2, 3).

**Table 2:** Descriptive statistics for self-harm.

**Date range**	**Time period**	**Total**	**Monthly mean**	**SD**	^*^% change	**Shapiro-Wilk (p)**
Jul 2017–Jun 2018	Pre-TIC	93	7.75	4.07	-	0.14
Jul 2018–Jun 2022	Post-TIC	164	3.42	3.95	↓ 55.91%	^*^0.00

* Non-normally distributed.

**Table 3:** Mann Whitney *U* comparison for monthly incidents of self-harm between one-year pre-TIC (July 2017–June 2018) and four-years post-TIC (July 2018–June 2022).

**Homogeneity of variance**		**Mann-Whitney** ***U***			**Effect size**
**F**	**Sig**.	**U**	**Z-score**	**Sig**.	**r**
1.06	0.41	464.5	3.28	0.00	^*^0.42

* Very large effect size.

**Figure 1:**
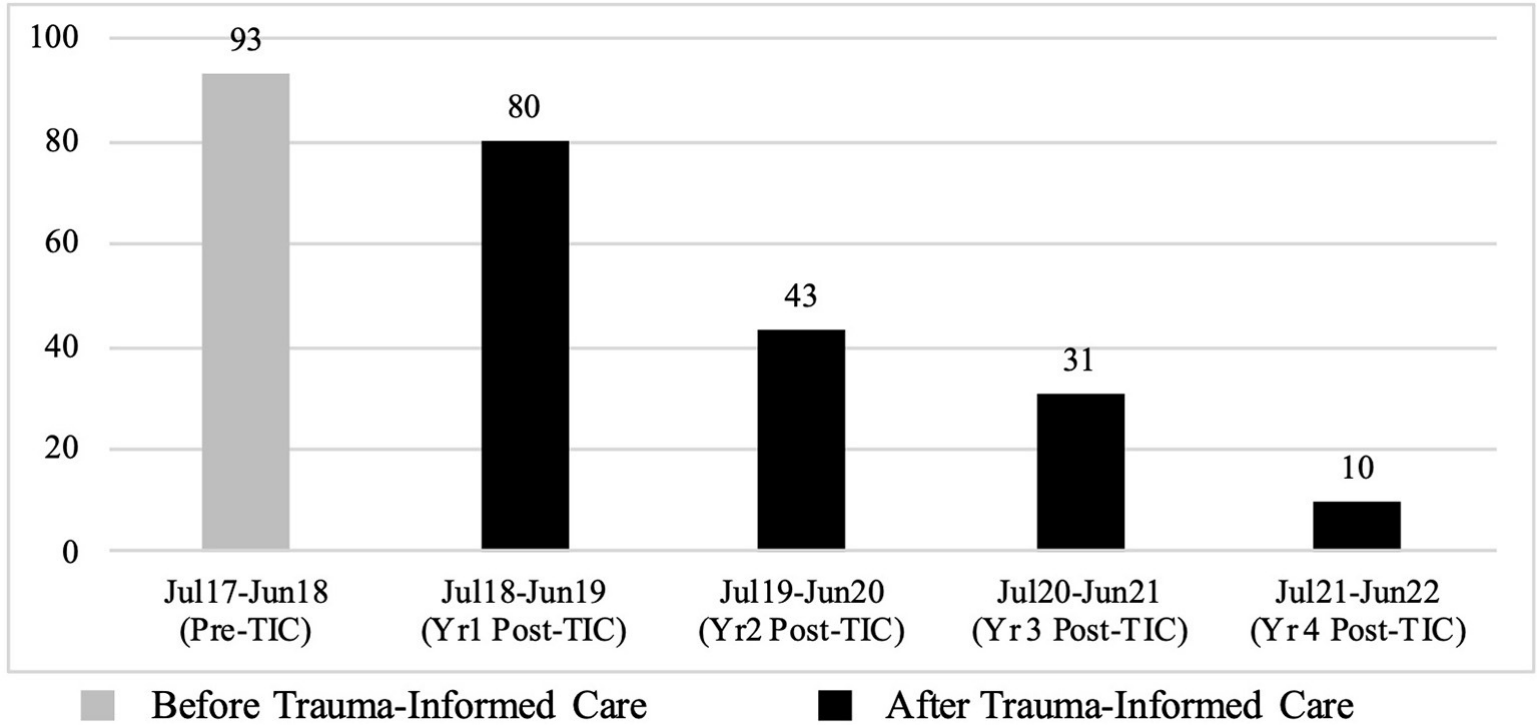
Total number of self-harm incidents between July 2017 and June 2022.

When the four-year period post-TIC is broken down by year, a continuously reducing trend is seen in the number of incidents of self-harm when compared to the year pre-TIC. This reduction ranges from 13.98% in the first year post-TIC to 89.25% in the fourth year post-TIC. See Figure 1 and Table 4 for further detail.

**Table 4:** Descriptive statistics for self-harm broken down by year.

**Date range**	**Time period**	**Total**	**Monthly mean**	**SD**	^*^% change
Jul 2017–Jun 2018	Pre-TIC	93	7.75	4.07	-
Jul 2018–Jun 2019	Year 1 Post-TIC	80	6.67	5.03	13.98% ↓
Jul 2019–Jun 2020	Year 2 Post-TIC	43	3.58	3.00	53.76% ↓
Jul 2020–Jun 2021	Year 3 Post-TIC	31	2.58	3.37	66.67% ↓
Jul 2021–Jun 2022	Year 4 Post-TIC	10	0.83	1.11	89.25% ↓

* Percentage of change in the monthly mean.

### 3.5. Restrictive interventions

#### 3.5.1. Seclusion

There was a total of 474 incidents of seclusion across the five-year period of evaluation (July 2017 - June 2022). 123 of these occurred in the first year of the evaluation (pre-TIC) compared to 351 that occurred in the later four-year period (post-TIC). Reasons for seclusion were, distress behaviour (*n* = 214, 45.44%); physical violence/ assault (*n* = 155; 32.70%); aggression/ non-physical abuse (*n* = 51, 10.76%); attempt to abscond (*n* = 31, 6.54%); self-harm (*n* = 10, 2.11%); vandalism (*n* = 7, 1.48%); medication (*n* = 3, 0.64); and COVID-19 positive refusal to isolate (*n* = 3, 0.63%).

The mean number of monthly incidents of seclusion in the year pre-TIC was 10.25. In comparison, there was a mean of 7.31 incidents of seclusion a month in the four-year period post-TIC. This represents an overall reduction of 28.66% in the use of seclusion post-TIC. Statistical analysis suggests this reduction to be significant with large effect size (see Tables 5, 6).

**Table 5:** Descriptive statistics for seclusion.

**Date range**	**Time period**	**Total**	**Monthly mean**	**SD**	^*^% change	**Shapiro-Wilk (p)**
Jul 2017–Jun 2018	Pre-TIC	123	10.25	3.72	-	0.14
Jul 2018–Jun 2022	Post-TIC	351	7.31	3.28	↓ 28.66%	^*^0.02

* Non-normally distributed.

**Table 6:** Mann Whitney U comparison for monthly incidents of seclusion between one-year pre-TIC (July 2017–June 2018) and four-years post-TIC (July 2018–June 2022).

**Homogeneity of Variance**		**Mann-Whitney** ***U***			**Effect size**
**F**	**Sig**.	**U**	**Z-score**	**Sig**.	**r**
1.29	0.26	415	2.35	0.02	^*^0.30

* Large effect size.

**Figure 2:**
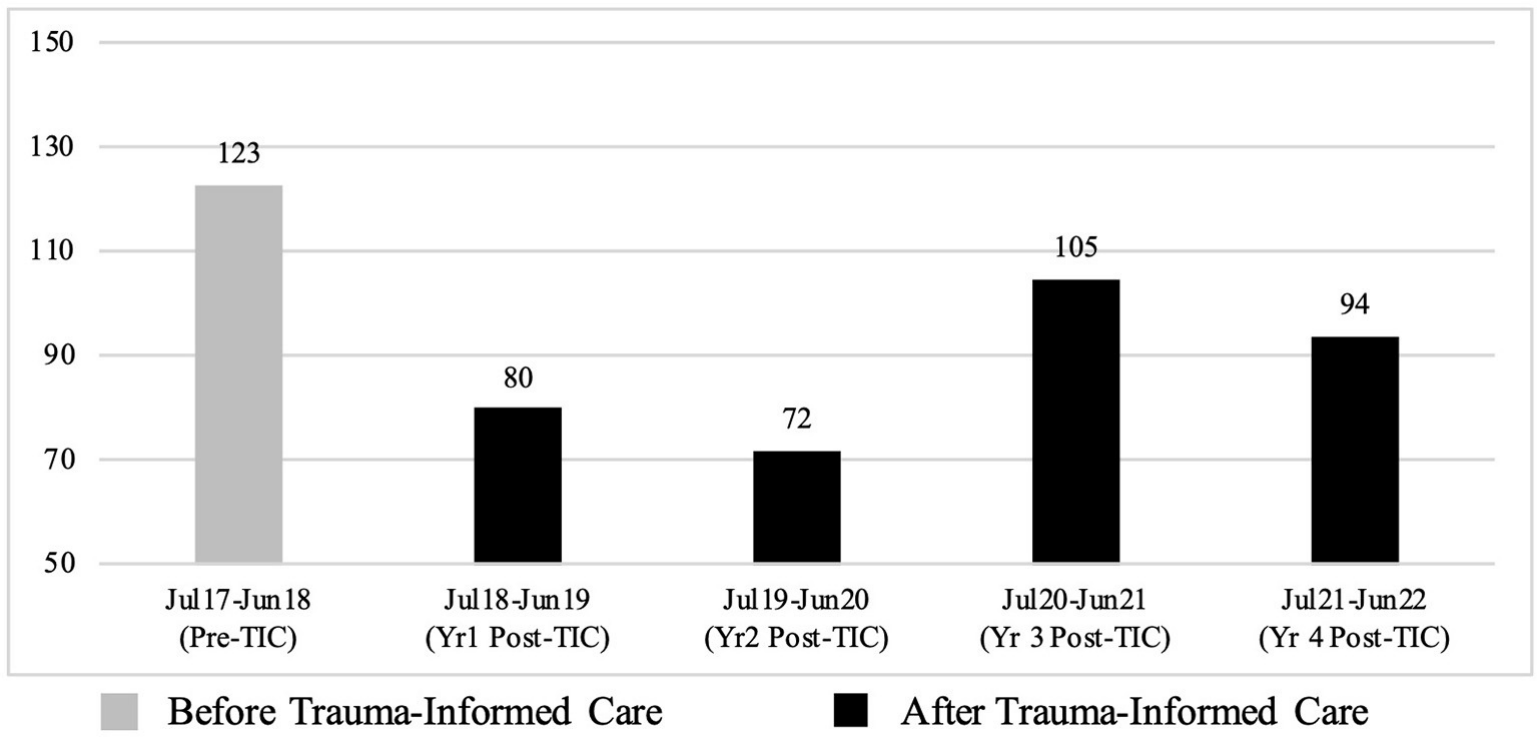
Total number of seclusion between July 2017 and June 2022.

When the four-year period post-TIC is broken down by year, reductions in the number of incidents of seclusions are seen for four of the four subsequent years and range from 14.63% (Year 3 post-TIC) to 41.46% (Year 2 post-TIC). An increase in the number of incidents of seclusion is seen in Year 3 post-TIC (July 2020 to June 2021) when compared to the previous two years post-TIC. Incidents reduce again in Year 4 but remain higher than for Years 1 and 2 post-TIC (Figure 2, Table 7). This spike in the seclusion data corresponds with large increases in staff sickness as a result of COVID-19 (see Section 3.5.3. Staff sickness for further detail).

**Table 7:** Descriptive statistics for seclusion broken down by year.

**Date range**	**Time period**	**Total**	**Monthly mean**	**SD**	^*^% change
Jul 2017–Jun 2018	Pre-TIC	123	10.25	3.72	-
Jul 2018–Jun 2019	Year 1 Post-TIC	80	6.67	2.90	34.96% ↓
Jul 2019–Jun 2020	Year 2 Post-TIC	72	6.00	3.30	41.46% ↓
Jul 2020–Jun 2021	Year 3 Post-TIC	105	8.75	3.39	14.63% ↓
Jul 2021–Jun 2022	Year 4 Post-TIC	94	7.83	3.19	23.58% ↓

* Percentage of change in the monthly mean.

#### 3.5.2. Restraint

There was a total of 812 incidents of restraint over the five-year evaluation period. 193 of these occurred in the first year of the evaluation (pre-TIC) compared to 619 in the four-year period post-TIC. Reasons for restraint were, distress behaviour (*n* = 348, 42.86%); physical assault (*n* = 251, 30.91%); attempt to abscond (*n* = 56, 6.90%); self-harm (*n* = 50, 6.16%); aggression/ non-physical abuse (*n* = 47, 5.79%); medication (*n* = 47, 5.79%); vandalism (*n* = 7, 0.85%); COVID-19 positive refusal to isolate (*n* = 3, 0.37%); and other (*n* = 3, 0.37%).

The mean number of monthly restraints in the year pre-TIC was 16.08. This was compared to a monthly mean of 12.90 restraints in the four-year period post-TIC. This represents an overall reduction of 19.82% in the use of restraint post-TIC. Statistical analysis (see Tables 8, 9) suggests this reduction to be significant with medium effect size.

**Table 8:** Descriptive statistics for restraint.

**Date range**	**Time period**	**Total**	**Monthly mean**	**SD**	^*^% change	**Shapiro-Wilk (p)**
Jul 2017–Jun 2018	Pre-TIC	193	16.08	5.66	-	0.16
Jul 2018–Jun 2022	Post-TIC	619	12.90	5.85	↓ 19.82%	0.27

* Percentage of change in the monthly mean.

**Table 9:** T-test for monthly incidents of restraint between one-year pre-TIC (July 2017–June 2018) and four-years post-TIC (July 2018–June 2022).

**Homogeneity of Variance**		**Independent** ***t*****-test**			**Effect size**
**F**	**Sig**.	**t**	**df**	**Sig**.	**Cohen's d**
0.91	0.46	1.68	58	0.049	^*^0.55

* Medium effect size.

**Figure 3:**
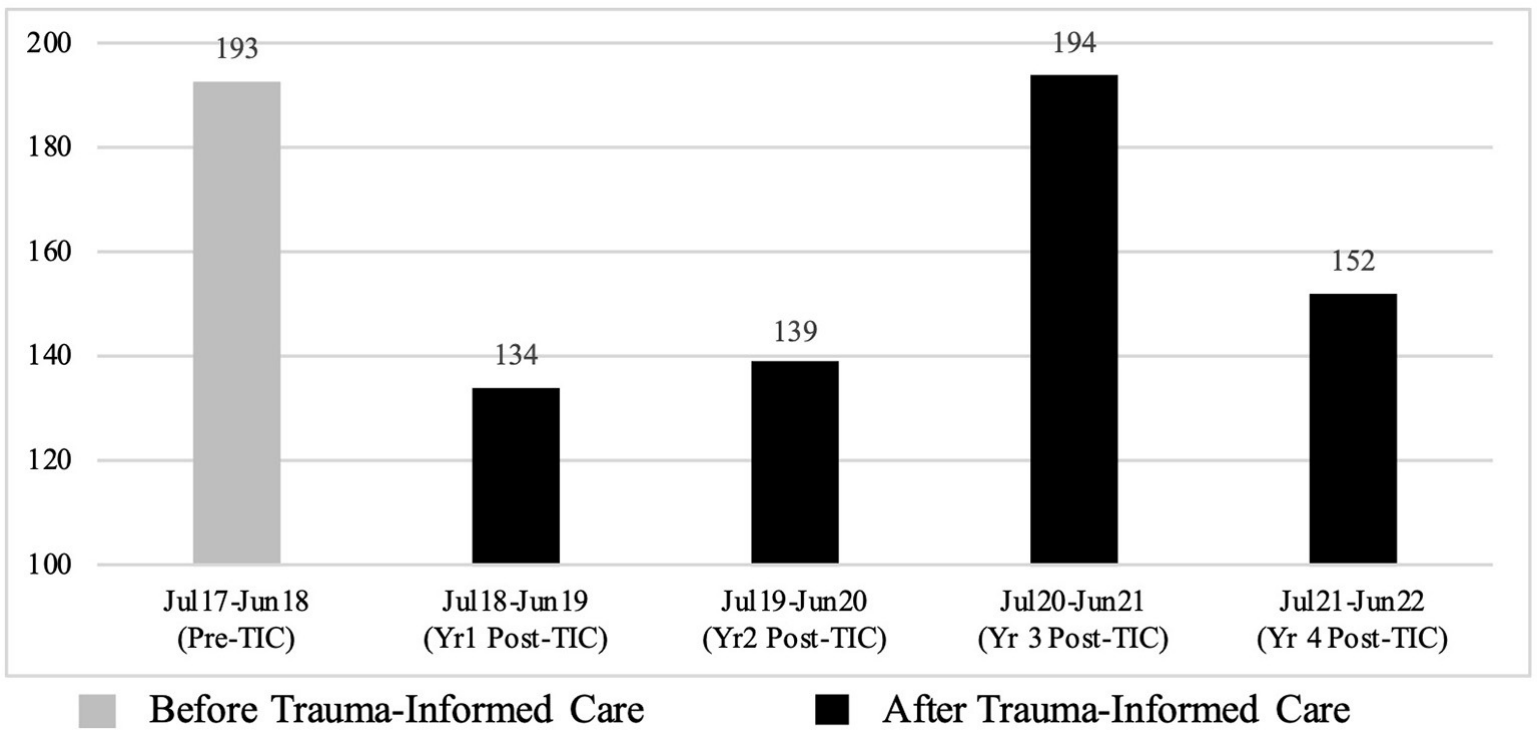
Total number of restraints between July 2017 and June 2022.

When the four-year period post-TIC is broken down by year, reductions in the number of incidents of restraint are seen for three of the four years, which range from 21.24% to 30.57% (see Figure 3 and Table 10). As with seclusion, after an initial reduction in the number of incidents of restraint for Years 1 and 2 post-TIC, there is an increase in the number of restraints in Year 3 post-TIC. This increase matches pre-TIC levels. Incidents reduce again in Year 4 post-TIC but remain higher than the original reductions made in Years 1 and 2 (Figure 3, Table 10). As with seclusion, this spike in the restraint data corresponds with large increases in staff sickness as a result of COVID-19 (see Section 3.5.3. Staff sickness for further detail).

**Table 10:** Descriptive statistics for restraint broken down by year.

**Date range**	**Time period**	**Total**	**Monthly mean**	**SD**	^*^% change
Jul 2017–Jun 2018	Pre-TIC	193	16.08	5.66	-
Jul 2018–Jun 2019	Year 1 Post-TIC	134	11.17	4.88	30.57% ↓
Jul 2019–Jun 2020	Year 2 Post-TIC	139	11.58	5.74	27.98% ↓
Jul 2020–Jun 2021	Year 3 Post-TIC	194	16.17	6.52	0.52% ↑
Jul 2021–Jun 2022	Year 4 Post-TIC	152	12.67	5.93	21.24% ↓

* Percentage of change in the monthly mean.

#### 3.5.3. Staff sickness

**Figure 4:**
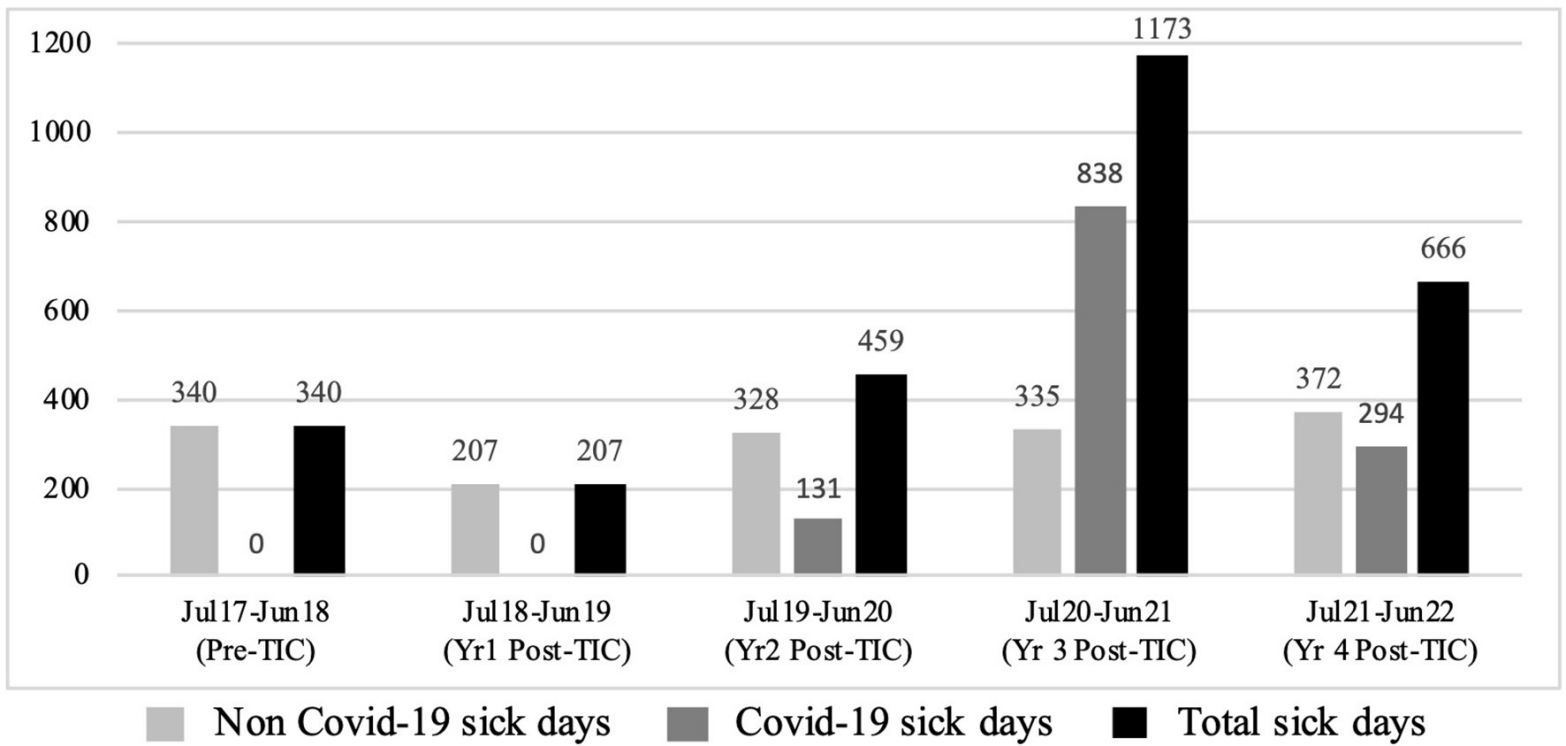
Total number of staff sick days.

The COVID-19 pandemic fell over the latter half of the evaluation period (Years 3 and 4 post-TIC) where an increase was seen in restrictive interventions (when compared to previous reductions found in Years 1 and 2 post-TIC). In line with this, there were extremely high numbers of staff sick days, directly related to COVID-19 (categorised as either, a: staff COVID-19 positive, or b: staff isolating due to household member with COVID-19) in Years 3 and 4 post-TIC. In comparison, staff sick days that were not directly related to COVID-19 remained relatively stable over the evaluation period (see Figure 4 and Table 11).

**Table 11:** Staff sick days.

**Date range**	**Time period**	**Non COVID-19 sick days**	**COVID-19 sick days**	**Total**	^*^% change
Jul 2017 - Jun 2018	Pre-TIC	340	0	340	-
Jul 2018 - Jun 2019	Year 1 Post-TIC	207	0	207	39.12% ↓
Jul 2019 - Jun 2020	Year 2 Post-TIC	328	131	459	35.00% ↑
Jul 2020 - Jun 2021	Year 3 Post-TIC	335	838	1,173	245.00% ↑
Jul 2021 - Jun 2022	Year 4 Post-TIC	372	294	666	95.88% ↑

* Percentage of change in staff sick days when compared to the year pre-TIC.

When COVID-19 sick days are included in the overall staff sickness data, a 245% and 95.88% increase in staff sickness can be seen in Years 3 and 4 post-TIC respectively (when compared to the year pre-TIC). These increases in staff sick days as a result of COVID-19 correspond with spikes seen in the restrictive interventions data in Years 3 and 4 post-TIC. With the easing of COVID-19 sick days (Years 4 post-TIC), the trend toward reduced levels of restraint and seclusion is re-established. Reasons for this are considered in the Discussion.

## 4. Discussion

This retrospective service evaluation aimed to assess the impact of introducing Trauma-Informed Care (TIC) to an NHS adult acute inpatient mental health unit in North London. This model of TIC comprised two trauma-informed practices, Power Threat Meaning Framework (PTMF) Team Formulation and Psychological Stabilisation training, which were introduced over a four-year period. Results indicate that these practices had a positive impact and contributed to significant reductions in incidents of self-harm (*p* < 0.01; very large effect size), seclusion (p < 0.05; large effect size) and restraint (*p* < 0.05; medium effect size) seen at the unit over this period. Fluctuations in these trends may be due to the impact of COVID-19 on staff sickness and related factors (as discussed in more detail below).

### 4.1. Self-harm

Outcomes in relation to self-harm were extremely positive, and the number of incidents reduced consecutively each year following the introduction of TIC. In the final year of the evaluation, incidents of self-harm had reduced by almost 90%. A more pronounced reduction in the number of incidents of self-harm was shown from years two (July 2019 – June 2020) onwards and this may suggest that a period of embedding of TIC was required.

It is acknowledged that improved psychology pathways, to support the transition of service users from inpatient to community services (from May 2020 onwards) as well as the development of a community Complex Emotional Needs pathway, to support individuals in the borough presenting with self-harm and suicidality associated with impulsivity (from May 2021 onwards) may have also contributed to the continued and sustained reduction in incidents of self-harm on the inpatient wards. Further evaluation will be necessary to better understand the relative contribution of these components.

To date, there has been limited research investigating TIC and self-harm behaviour. However, findings are in line with wider research showing that TIC supports service users to develop coping skills (Gatz et al., 2007); reduce levels of distress/ symptoms (Messina et al., 2014); and improve mental wellbeing (Greenwald et al., 2012).

### 4.2. Restrictive intervention

Outcomes in relation to restrictive interventions (seclusion and restraint) were also positive and overall, significant reductions were shown in both areas. This finding supports previous research by Azeem et al. (2011) that also demonstrates a reduction in restrictive interventions associated with TIC. When the data was broken down by year, reductions in seclusion and restraint were demonstrated for the first two years following the introduction of TIC. Interestingly, these reductions were not maintained at year three. In the fourth year following the introduction of TIC, incidents of restraint and seclusion reduced again, but not to the same levels seen in years one and two.

Data on the very high levels of staff sickness absence as a result of the COVID-19 pandemic (which fell over the latter phase of the evaluation period) suggest that this may account for the spike in restrictive interventions. In years three and four post-TIC, staff sick days increased by 245% and 95.88% respectively, compared with the year prior to TIC. These sharp rises in sickness were attributable to COVID-19 absences (either, a: staff COVID-19 positive, or b: staff isolating due to household member with COVID-19) and non COVID-19 related sickness remained relatively stable across the duration of the evaluation period. When staff are off sick, shifts are covered by bank or agency staff, who are not trained in and would not have been engaging with the unit's trauma-informed practices. Permanent staff may also be required to work overtime or shifts may run with staff-shortages. In addition, research has shown that working through the COVID-19 pandemic negatively impacted the mental health and wellbeing of frontline staff significantly (Gilleen et al., 2021). All of these variables could have influenced the unit's capacity to maintain their reduced levels of restrictive intervention.

### 4.3. Mechanisms of change

Several hypotheses are put forward to explain the mechanisms by which the two evaluated trauma-informed practices may have contributed to the overall reductions found in self-harm, restraint and seclusion.

#### 4.3.1. PTMF Team Formulation

Research has shown Team Formulation to be an effective tool in supporting staff teams to develop a shared understanding of the service user (Geach et al., 2018). The model of Team Formulation presented in this paper employed the PTMF to support a trauma-informed version of this practice. The shared understanding developed by staff in Team Formulation meetings can support the generation of new ideas, intervention planning and improved safety management (Hollingworth and Johnstone, 2014). It is therefore proposed that PTMF Team Formulation may have contributed to the reductions in self-harm and restrictive intervention by supporting the inpatient team to better understand service users from a trauma-informed perspective and develop novel ways of approaching distress on the ward. By making explicit service users' past experiences of trauma, and their meaningful links to current behaviour, PTMF Team Formulation may have also increased staff's understanding of the potential for re-traumatisation (Sweeney et al., 2016) and the importance, where possible, for creative alternatives to restrictive intervention.

Additionally, research has shown Team Formulation to strengthen relationships between staff and service users (Berry et al., 2015); increase levels of empathy toward service users (Whitton et al., 2016); and improve teamwork and communication between staff (Kramarz et al., 2022). All of these factors could also have positively influenced the reductions seen in self-harm and restrictive intervention at the unit. Qualitative interviews with both staff and service users, which are currently being prepared for publication, suggest support for these possibilities.

#### 4.3.2. Stabilisation

The Stabilisation Manual comprised interventions that have been shown to support service users to better manage emotional dysregulation (Neacsiu et al., 2014), unusual experiences (Chadwick et al., 2005) and self-harm (Kothgassner et al., 2021). It is suggested that training staff to deliver these interventions may have contributed to the reductions seen in self-harm and restrictive intervention in several ways. Firstly, overall levels of service users' distress, which can result in incidents of self-harm or incidents requiring staff to intervene with restrictive intervention, may have been reduced. Secondly, service users may have felt more able to cope with distress so as not to result in incidents of self-harm or the need for restrictive intervention. And finally, when incidents did occur on the ward, staff may have been more able to respond in ways that prevented an escalation to self-harm or restrictive intervention. As above, qualitative interviews with both staff and service users, which are currently being prepared for publication, suggest support for these possibilities.

### 4.4. Strengths and limitations

Strengths of this project include, the development of a standardised model for TIC (comprising two practices) that could easily be replicated in other settings; the positive impact of these practices in reducing incidents of self-harm and restrictive interventions in an acute inpatient mental health setting; and the engagement of the full multi-disciplinary team. Strengths of the evaluation include, the length of the evaluation period (with four-years of post-implementation data); and the naturalistic NHS setting that provides increased ecological validity. Recent media publicity about coercion on inpatient wards (e.g., Dispatches, 2022; Panorama, 2022) indicates an urgent need to find ways of reducing coercive and potentially re-traumatising interventions. The model described here suggests one such approach.

Limitations of the project include its lack of formal coproduction. Although service users were consulted within the development of this project, there was no service user representation in the team of Champions that led on the development and facilitation of the two trauma-informed practices (PTMF Team Formulation and Psychological Stabilisation). Additional funding for Advanced Lived Experience Practitioners (senior staff with lived experience) for the mental health unit has since been approved and it is hoped this group will comprise a key element to the team of Champions leading on TIC for the unit moving forward. It is also acknowledged that there was a lack of medical staff involvement in the development and facilitation of the practices, and this will be another area of focus for future work.

Limitations of the evaluation include, the retrospective service evaluation design and lack of control group, which increase the risk of Type 1 Error and mean that reported effects cannot conclusively be attributed to this model of TIC. Nor can we draw conclusions about the relative contribution of each of the core aspects of the approach, although qualitative data (currently being prepared for publication) suggests that both were seen as complementary and important. It is also noted that the reduction in inpatient beds over the evaluation period could have influenced results. However, data showing relative consistency in the number of admissions over this period (despite the change in bed base) seems to contradict this conclusion.

### 4.5. Clinical application

The NHS Long Term Plan (NHS, 2019) has laid out recommendations for services to be trauma-informed over the next 10 years. However, as yet there are no standardised models to support services to embed this. The two trauma-informed practices put forward in this evaluation could represent one possible approach toward meeting this aim. The model of TIC described in this paper is easily transferable and alongside acute inpatient mental health settings could also be delivered in crisis and community mental health services. This will constitute the next stage of the project and pilots have been commenced in local teams.

### 4.6. Future research

This paper is part of a larger evaluation investigating the impact of this model of TIC and its two practices of PTMF Team Formulation and Psychological Stabilisation. Due to the scope of this paper, only the quantitative data is presented here. Qualitative interviews with service users and staff, which are being prepared for future publication, will support a richer understanding of the impact of these practices.

Future research might also evaluate the impact of these trauma-informed practices in a randomised control trial across several inpatient mental health units, which would increase the generalisability and validity of findings. A larger data set generated by the inclusion of multiple trial sites could facilitate superior statistical modelling and control for confounding variables. However, the ethical implications of an experimental design, where individuals in acute psychological distress are randomised to a control group without access to potentially beneficial practice, may outweigh the benefits of such a study.

Based on this preliminary data, 13 other adult inpatient mental health wards in this NHS Trust are now implementing this approach (at various stages of development) with support from the authors. Data from these boroughs may be available at a future point. The impact of these trauma-informed practices in other settings could also be investigated.

## 5. Conclusion

This service evaluation outlines a novel model of Trauma-Informed Care (TIC), comprising Power Threat Meaning Framework (PTMF) Team Formulation and Psychological Stabilisation training. Findings suggest that the implementation of this model can contribute to significant and sustained reductions in incidents of self-harm, seclusion and restraint in an adult inpatient mental health setting, and highlight the benefits of TIC in this context.

## Data availability statement

The raw data supporting the conclusions of this article will be made available by the authors, without undue reservation.

## Ethics statement

Ethical review and approval was not required for the study on human participants in accordance with the local legislation and institutional requirements. Written informed consent for participation was not required for this study in accordance with the national legislation and the institutional requirements.

## Author contributions

FN and GB contributed to the conception, administration, and supervision of this project. JC and AS contributed to the project administration and data curation. FN performed the analysis and wrote the manuscript. All authors contributed to the article and approved the submitted version.
