# Supplementary material for: Trauma-Informed Care on mental health wards: the impact of Power Threat Meaning Framework Team Formulation and Psychological Stabilisation on self-harm and restrictive interventions
Source: Front Psychol. 2023 Jun 8;14:1145100. doi: 10.3389/fpsyg.2023.1145100 (PMC10285464; doi:10.3389/fpsyg.2023.1145100)
Supplement: Supplementary file 1 [file Data_Sheet_1.docx]

# Appendix 1 - Power Threat Meaning Framework Team Formulation Protocol

**Section 1: Introduction (approximately 15-minutes)**

**Background review**

Facilitators introduce staff and set the frame for the meeting. Following this, a member of the team presents a summary of the service user’s background and history, based on a simple template developed for this purpose. Although not present at the meeting, where possible the service user is informed of the meeting, involved in the preparation of material and asked for their views and feedback.

**Feelings**

Staff are asked to reflect on their feelings in relation to the service user. Facilitators model that it is okay to name more difficult emotions and make it safe for staff to bring a range of feelings, as long as they are taken ownership of and blaming statements about the service user are avoided.

**Stuck points**

Staff are asked to think about where they are feeling stuck in relation to the service user. This purposely positions the problem with the team rather than focusing on the service user as the ‘problem’ or sole agent for change.

**Section 2: Formulation (approximately 30-minutes)**

**Power resources**

The formulation starts by sharing information about the service user’s strengths and resources to support the team to develop a more hopeful and holistic understanding of the person behind the medical labels.

**Power imbalances**

Experiences of trauma and adversity that the service user has experienced in the context of power imbalance (for example; racial discrimination, sexual abuse) are discussed. Imbalance is considered at multiple levels (individual, familial, societal, structural) and facilitators identify and emphasise repeated patterns of adversity throughout life (such as, repeated experiences of loss). Power imbalances perpetuated by the mental health system are acknowledged (for example; restrictive intervention).

**Threat**

The impact of adversity is considered in terms of how it may have threatened the service user’s core safety, survival and wellbeing needs. For example, how experiences of abuse may threaten one’s physical, relational and emotional safety.

**Meaning**

The team reflects on how the service user may have made sense of their experiences, based both on what they have explicitly said as well as the tentative generating of hypotheses by the team. Facilitators support explicit links between power and threat to ensure the concept of meaning is not de-contextualised. For example, making clear links between ‘delusional’ beliefs a service user may hold about other people as dangerous, and their past experiences of abuse and unsafety.

**Threat responses**

Symptoms are re-formulated as threat responses, and facilitators support the team to make explicit links to earlier parts of the formulation. For example, making links between experiences of unsafety, potential beliefs about others as dangerous, and a threat response of aggression, which may in turn result in further threats to that service user’s safety.

**Section 3: Close (approximately 15-minutes)**

**Feelings**

The team’s feelings that were generated at the start are revisited. The team is supported to better understand these feelings in the context of the formulation and shifts in staff feelings are acknowledged.

**Ways forward**

The meeting concludes with the team thinking together about how to move forwards, using the formulation as a map. This may include conveying the provisional thoughts of the team to the service user in an appropriate way, so that their views can feed into the ongoing formulation process.
